# Comprehensive analysis of gut microbiome and host transcriptome in chickens after *Eimeria tenella* infection

**DOI:** 10.3389/fcimb.2023.1191939

**Published:** 2023-06-06

**Authors:** Hailiang Yu, Qi Wang, Jianqiang Tang, Liyue Dong, Guojun Dai, Tao Zhang, Genxi Zhang, Kaizhou Xie, Hongsheng Wang, Zhenhua Zhao

**Affiliations:** ^1^ College of Animal Science and Technology, Yangzhou University, Yangzhou, China; ^2^ Technical Research Department, Jiangsu Jinghai Poultry Group Co., Ltd., Haimen, China; ^3^ Poultry Institute, Chinese Academy of Agricultural Sciences, Yangzhou, China

**Keywords:** chicken, *E. tenella*, cecal microbiome, transcriptome, integrated analysis

## Abstract

**Background:**

Coccidiosis is an intestinal parasitic disease caused by *Eimeria* protozoa, which endangers the health and growth of animals, and causes huge economic losses to the poultry industry worldwide every year. Studies have shown that poultry gut microbiota plays an important role in preventing the colonization of pathogens and maintaining the health of the host. Coccidia infection also affects host gene expression. However, the underlying potential relationship between gut microbiome and host transcriptome during *E. tenella* infection in chickens remain unclear.

**Methods:**

In this study, metagenomic and transcriptome sequencing were applied to identify microbiota and genes in cecal contents and cecal tissues of infected (JS) and control (JC) chickens on day 4.5 postinfection (pi), respectively.

**Results:**

First, microbial sequencing results of cecal contents showed that the abundance of *Lactobacillus, Roseburia* sp. and *Faecalibacterium* sp decreased significantly after *E. tenella* infection (*P* < 0.05), while the abundance of *Alistipes* and *Prevotella pectinovora* increased significantly (*P* < 0.05). Second, transcriptome sequencing results showed that a total of 434 differentially expressed mRNAs were identified, including 196 up-regulated and 238 down-regulated genes. These differentially expressed genes related to inflammation and immunity, such as *GAMA, FABP1, F2RL1 and RSAD2*, may play an important role in the process of host resistance to coccidia infection. Functional studies showed that the enriched pathways of differentially expressed genes included the TGF-beta signaling pathway and the ErbB signaling pathways. Finally, the integrated analysis of gut microbiome and host transcriptome suggested that *Prevotella pectinovora* associated with *FABP1*, *Butyricicoccus* porcorum and *Colidextribacter* sp. associated with *RSAD2* were involved in the immune response upon *E. tenella* infection.

**Conclusion:**

In conclusion, this study provides valuable information on the microbiota and key immune genes after chicken *E. tenella* infection, with the aim of providing reference for the impact of coccidia infection on cecal microbiome and host.

## 1 Introduction

Coccidiosis is a poultry intestinal parasitic disease caused by *Eimeria* protozoa, which seriously affects the development of the global poultry industry. Studies have shown that the annual global economic loss due to coccidiosis exceeds $14 billion (Blake et al., 2020; Wickramasuriya et al., 2023). Economic costs mainly include the prevention and treatment of coccidiosis (coccidiosis drugs and high vaccine costs) and subclinical symptoms of chicken populations. Infected chickens cause enormous economic losses to the poultry industry, including reduced body weight gain, feed conversion and egg production (Burrell et al., 2019; Yu et al., 2020). At present, due to the prohibition of antibiotics and food safety issues, the use of traditional anticoccidial drugs is limited and the alternative strategies for coccidiosis require a lot of attention (Yu et al., 2021a).

The gastrointestinal system of poultry, like mammals, is a complex physiological structure that plays crucial roles in digestion and host health. The gut microbiota is able to digest food ingested by animals and provide essential nutrients for growth, development, reproduction and daily activities of the organism (Baldwin et al., 2018; Madlala et al., 2021). In addition, when pathogens invade, gut microbiota can inhibit the colonization of pathogens and play an important role in maintaining intestine homeostasis and host health (Stanley et al., 2014; Clavijo and Flórez, 2018). Compared to other segments of the gut, such as duodenum, jejunum and ileum, the cecum has the highest number, richness and diversity of gut microbiota (Yeoman et al., 2012; Rubio, 2019), which play an important role in the repeated fermentation and absorption of nutrients. *E. tenella* mainly invades the cecal tissue of chickens, leading to swelling and bleeding of the cecum tissues and changes of cecal microbiota, which ultimately reduce the production performance of chickens (Yu et al., 2022). Studies have shown that gut microbiota play an important role in the resistance of poultry to coccidia infections. Butyrate alleviates the severity of *E. tenella* infection in chickens (Chen et al., 2020). Li et al. (2017) showed that the addition of *Lactobacillus acidophilus* to the diet enhanced the inflammatory response in poultry and modulated the innate and acquired immune response. In addition, in ovo supplementation with probiotics protected early chicks against *Eimeria* infection (Pender et al., 2016). Therefore, microbiota plays an important role in birds’ resistance to pathogens such as coccidiosis and maintaining healthy growth and development.

In recent years, the development of high-throughput sequencing technologies and omics analysis have significantly expanded our knowledge of the gut microbiota of animals. However, the detailed function of the gut microbiota in infected diseases (such as coccidiosis) and the interactions between the microbiota and the host remain unclear. In this study, the metagenomic and transcriptome sequencing technologies were used to sequence the cecal contents and cecal tissue of infected group and control group chickens on day 4.5 pi to explore the effect of coccidia infection on cecal microbiota and host gene expression, respectively and interaction analysis between microbiota and host was also carried out. The purpose of this study is to provide a reference for the impact of coccidia infection on cecal microbiome and host.

## 2 Materials and methods

### 2.1 Animals and oocysts

A total of twenty 1-day-old healthy Chinese yellow-feathered broiler chicks with similar size were randomly selected. The experimental chicks were kept in a clean, pathogen-free animal room, with free access to food and water. At the age of 18 days, all the chicks were divided into two groups. Each bird in the infection group was orally fed with 3.5×10^4^ sporulated oocysts (water was not provided within 6 hours after the challenge to ensure that the oocysts were ingested by the birds), and the chicks of control group were fed with the same amount of normal saline. *E. tenella* oocysts, a Yangzhou strain in China, were a gift from the Parasite Laboratory in the Veterinary Medicine College of Yangzhou University. The collection, purification and sporulation of fresh oocysts were described in previous articles (Yu et al., 2022).

### 2.2 Sample collection

On 4.5 days (108 h) post infection, 4 individuals of female chicks were randomly selected from each group and euthanized by rapid cervical dislocation. After the cecum was isolated, the intestinal contents were collected immediately in cryovials. The cecum tissues were washed with sterilized PBS for excess intestinal contents and mucus, and placed in cryopreservation tubes. All samples were immediately stored in liquid nitrogen and then transferred to a -80°C refrigerator for later use. The animal experiment protocols were approved by the Animal Welfare Committee of Yangzhou University (permit number: SYXK (Su) IACUC 2012-0029).

### 2.3 Metagenomic sequencing analysis of cecal microbiome

#### 2.3.1 DNA isolation and library construction

Total DNA of chicken cecal microorganisms was extracted using a QIAamp® Fast DNA Stool Mini Kit (Qiagen, Hilden, Germany) according to the manufacturer’s instructions. The DNA integrity of the sample was detected by agarose gel electrophoresis, and the DNA concentration was measured with a NanoDrop2000 spectrophotometer (Thermo Fisher Scientific, Waltham, MA, USA). Library construction was performed using the TruSeq Nano DNA LT Sample Preparation Kit (Illumina, San Diego, CA, USA). The library construction and sequencing were carried out by OE Biomedical Technology Co., Ltd (Shang hai, China).

#### 2.3.2 Metagenome sequencing analysis

The library was sequenced using the llumina Novaseq 6000 sequencing platform, and 150 bp paired-end reads were generated (the sequencing depth/volume is 10 G). The raw data is preprocessed using Trimmomatic (v 0.36) (Bolger et al., 2014), including removing adapters, low-quality Reads and low-quality bases; The filtered reads were aligned to the chicken genome (Gallus gallus bGalGal1.mat.broiler. GRCg7b) using bowtie2 (v 2.2.9), and the aligned reads were removed. Then Metagenome assembly was performed on the valid reads using the splicing software MEGAHIT (v 1.1.2) (Li et al., 2015). ORF prediction of assembled scaffolds using prodigal was performed and translated into amino acid sequences. The non-redundant gene sets (in-house version) were built for all predicted genes using CDHIT (v 4.5.7). The Clean reads of each sample were aligned against the non-redundant gene set (95% identity) use bowtie2 (v 2.2.9), and the abundant information of the gene in the corresponding sample was counted.

Species annotations were obtained through the corresponding taxonomic information database of NR library, and then the abundance of the species was comprehensively calculated using the corresponding abundance of gene. To construct the abundance profile of taxonomy level, abundance statistics of samples were performed at different level, including Domain, Kingdom, Phylum, Class, Order, Family, Genus and Species. The gene set representative sequence (amino acid sequence) was annotated with COG, NR, SWISSPROT, KEGG and GO database with an e-value of 1e-5 using DIAMOND (v 0.9.7) (Buchfink et al., 2015). The taxonomy abundance spectrum or functional abundance spectrum were subjected to PCA analysis and plotted using prcomp function and ggplot2 package in R.

#### 2.3.3 Predictive function of differential cecal microbiota

To reveal the predictive function of differential microbiota after coccidia infection, eggNOG (Evolutionary genealogy of genes: Non-supervised Orthologous Groups) and were used for pathway enrichment analysis of differential cecal microbiota. The eggNOG database used the Smith-Waterman alignment algorithm to annotate the function of constructed Orthologous Groups. KEGG database is a comprehensive database for annotating gene functions (DIAMOND software), the core of which is kegg pathway and kegg orthology database.

### 2.4 Transcriptome sequencing analysis

#### 2.4.1 RNA isolation

Total RNA from cecum tissues were extracted with TRIzol reagent (Invitrogen, CA, USA) following the manufacturer’s recommended procedure. The NanoDrop 2000 spectrophotometer (Thermo Fisher Scientific, Waltham, MA, USA) was used to measured RNA purity and concentration, and the Agilent 2100 Bioanalyzer (Agilent Technologies, Santa Clara, CA, USA) was used to evaluate RNA integrity. The Host transcriptome sequencing can be performed on the basis of meeting RNA quality standards (RNA integrity number>=7 and 28S/18S>=0.7).

#### 2.4.2 Library construction and Sequencing

Then the libraries were constructed using TruSeq Stranded Total RNA with Ribo-Zero Gold (illumina, Cat.No. RS-122-2301) according to the manufacturer’s instructions. The libraries were sequenced on an Illumina HiSeq X Ten platform, 150 bp paired-end reads were generated (the sequencing depth/volume is 6 G). Raw reads of fastq format were firstly processed using fastp and the low-quality reads were removed to obtain the clean reads. Then the clean reads were mapped to the chicken reference genome using HISAT2 (Kim et al., 2015). For mRNAs, FPKM (Roberts et al., 2011) of each gene was calculated using Cufflinks (Trapnell et al., 2010), and the read counts of each gene were obtained by HTSeq-count (Anders et al., 2015). Differential expression analysis was performed using the DESeq2 package in R. *P-value* < 0.05 was set as the threshold for significantly differential expression.

#### 2.4.3 Functional enrichment analysis of differentially expressed genes

After the differentially expressed genes were obtained, GO enrichment analysis was performed to describe their functions. GO enrichment contains three class: biological process, cellular component and molecular function. Moreover, the KEGG database was used to conduct pathway enrichment analysis of differential expressed genes. GO terms and KEGG pathways were considered to be significantly enriched based on a *P-value <*0.05.

### 2.5 Integrated analysis of gut microbiome and host transcriptome

To explore the potential relationship between cecal microbiome and hosts after *E. tenella* infection, Spearman correlation coefficient was used to calculate the correlation between differentially microbiota and differentially expressed genes. Spearman correlation coefficient |r| > 0.8 and *P-value* < 0.05 are considered as significantly relationship pairs. The correlation heatmap of cecal microbiota and differentially expressed genes were constructed using the R package, and the co-expression network map were constructed using Cytoscape (v3.8.2) (Smoot et al., 2011).

### 2.6 Correlation analysis of gut microbiome after *E. tenella* infection

Moreover, to explore the potential relationship between cecal microbiome and hosts after *E. tenella* infection, this study also selected the top 30 microbiotas with the total abundance at the genus level, and used the Spearman correlation coefficient to explore the correlation between dominant genus. The R package was used to generate the correlation heatmap of the dominant genus.

### 2.7 Histopathology

The cecal tissues from JC and JS groups were cut into approximately 0.5 cm pieces and fixed with 4% tissue fixative. After embedding in paraffin and sectioning (5 μm), the sections were stained with hematoxylin-eosin. Then, the slices were observed and imaged under a microscope (Nikon, Japan).

### 2.8 Statistical analysis

The difference between different groups was analyzed using Wilcoxon rank-sum test. The linear discriminant analysis effect size (LEfSe) method (Segata et al., 2011) was used to compare the taxonomy abundance spectrum or functional abundance spectrum and Linear discriminant analysis (LDA) score > 2.0 and *P-value <*0.05 were considered as significant difference. In addition, the correlation analysis was constructed to investigate the interaction between microbiome and host transcriptome, and Spearman coefficient |r| > 0.8 and *P-value* < 0.05 were shown.

## 3 Results

### 3.1 Chicken models of *E. tenella* infection

On day 4.5 pi, all chickens were euthanized and cecal tissues were harvested. As expected, the cecal tissues of the chickens in JS group had severe intestinal bleeding, and the cecum wall was obviously swollen and thickened, while the intestinal tissue structure of the chickens in JC group was intact (**Supplementary Figure S1**). In addition, the pathological analysis of cecal tissue in this study showed that the tissue layers of JC group chickens had a clear structure, the mucosal epithelium was intact, and the epithelial cells were tightly arranged. Extensive mucosal layer necrosis and ulcers appeared in the cecal tissue of JS group chickens (**Supplementary Figure S2**). The results of PCA plot also showed that the cecal microbiota of the JS group was different from that of the JC group (**Supplementary Figure S3**). Thus, these data indicated that the chicken model of *E. tenella* infection was successfully constructed.

### 3.2 Effects of *E. tenella* infection on cecal microbiota

**Figure 1:**
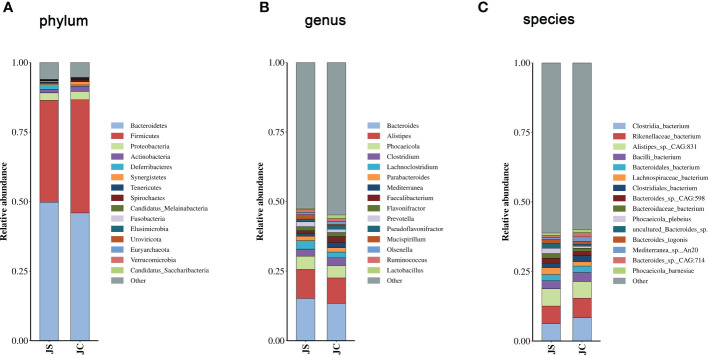
Comparison of microbial community composition between JC and JS groups. **(A)**, phylum. **(B)**, genus. **(C)**, species. JC, control group; JS, infected group.

To reveal the impact of *E. tenella* infection on host cecal microbiota, we analyzed the changes of cecal microbiota at the phylum, genus, and species level (**Figure 1**, **Supplementary Figure 4**). At the phylum level, *Bacteroidetes*, *Firmicutes*, *Proteobacteria* and *Actinobacteria* were the most dominant phylum in the chicken cecal contents (**Figure 1A**). Moreover, we found that the abundance of *Firmicutes*, *Proteobacteria* and *Actinobacteria* decreased, while the abundance of *Bacteroidetes* increased after *E. tenella* infection. At the genus level, *Bacteroides*, *Alistipes*, *Phocaeicola*, and *Clostridium* were the most dominant genus in the chicken cecal contents (**Figure 1B**). In addition, *Clostridia bacterium*, *Rikenellaceae bacterium*, *Alistipes* sp. *CAG:831*, and *Bacilli bacterium* at the species level had the relatively high abundance in chicken cecal microbiota (**Figure 1C**).

### 3.3 Differential abundance analysis of cecal microbiota after chicken *E. tenella* infection

**Figure 2:**
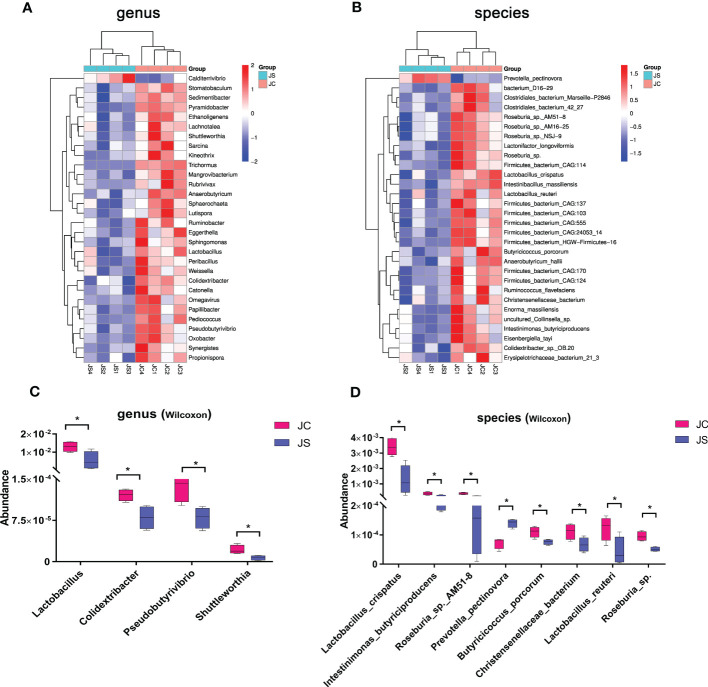
Differential abundance analysis of cecal microbiota after chicken *E. tenella* infection. **(A, B)** Hierarchical clustering plots of top 30 microbiota with significant differences at genus level **(A)** and species level **(B)**. **(C, D)** The relative abundance of key microbiota at genus level **(C)** and species level **(D)**. The bar represents the maximum and minimum values of a set of data. Statistical analyses were performed using Wilcoxon rank-sum test (*P < 0.05). JC, control group; JS, infected group.

To identify the important microbiota during the process of *E. tenella* infection in chickens, we performed differential abundance analysis of cecal microbiota in different groups by using the Wilcoxon rank-sum test. The results of the top 30 species with significant differences were shown in **Figure 2**. At genus level, *E. tenella* infection significantly reduced the abundance of *Lactobacillus*, *Colidextribacter*, *Pseudobutyrivibrio*, *Shuttleworthia*, and *Weissella* in the cecal microbial community (*P <*0.05, **Figure 2A, C**). At the species level, the abundance of *Lactobacillus reuteri*, *Lactobacillus crispatus*, *Intestinimonas butyriciproducens*, *Butyricicoccus porcorum*, and *Roseburia* sp. in the cecal microbiota were significantly reduced when chickens were infected by *E. tenella* (*P <*0.05, **Figure 2B, D**). However, the abundance of *Prevotella pectinovora* increased significantly (*P <*0.05, **Figure 2D**). Taken together, these results suggest that coccidiosis infection alters the composition of the cecal microbiota.

### 3.4 LEfSe analysis of differentially microbiota in chicken cecal contents after *E. tenella* infection

**Figure 3:**
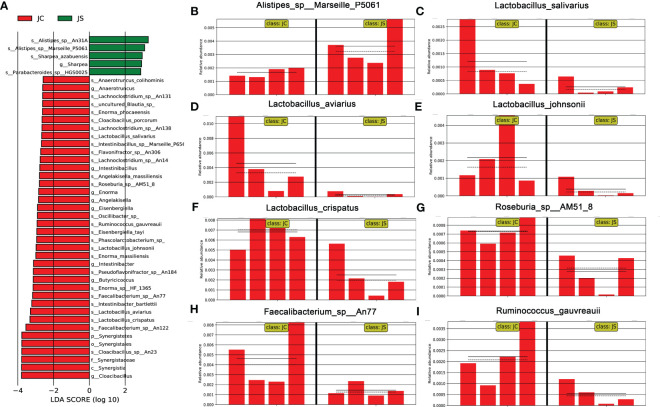
Linear discriminant analysis effect size (LEfSe) analysis of the cecal microbiota in different groups. **(A)** LDA score of differential cecal microbiota. LDA score > 2.0 and *P* < 0.05 were considered to be significantly differences. The green and red horizontal color bars represent microbial enrichment in the JS and JC groups, respectively. JC, control group; JS, infected group. **(B-I)** Histogram of relative abundance of differential cecal microbiota. **(B)** *Alistipes_sp:Marseille_P5061*. **(C)** *Lactobacillus_salivarius*. **(D)** *lactobacillus_aviarius*. **(E)** *lactobacillus_johnsonii*. **(F)** *lactobacillus_crispatus*. **(G)** *Roseburia_sp:AM51_8*. **(H)** *Faecalibacterium_sp:An77*. **(I)** *Ruminococcus_gauvreauii*.

LEfSe analysis, also known as LDA EFfect Size analysis, is an analytical method to count and find biomarkers with significant differences between different groups. To further understand and reveal the effects of *E. tenella* infection on the microbial community of chicken cecum, we performed LEfSe analysis of cecal microbiota in the infected and control chickens (**Figure 3A**). Similarly, we found that the abundance of *lactobacillus salivarius*, *lactobacillus aviarius*, *lactobacillus johnsonii*, and *lactobacillus crispatus* decreased during *E. tenella* infection (**Figures 3C-F**). In addition, the abundance of *Roseburia sp*, *Faecalibacterium sp* and *Ruminococcus gauvreauii* also decreased (**Figures 3G-I**). However, the abundance of *Alistipes* increased after coccidia infection (**Figure 3B**).

### 3.5 Functional enrichment analysis of differential microbiota after chicken *E. tenella* infection

**Figure 4:**
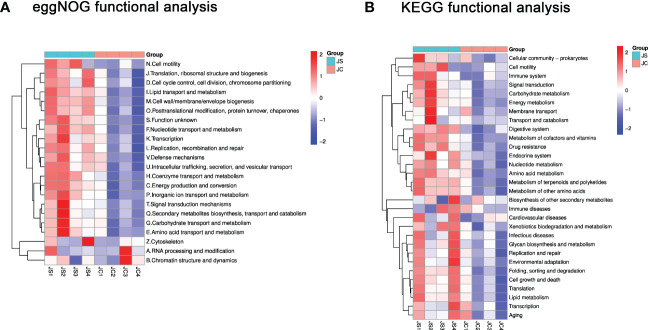
Functional enrichment analysis of differential cecal microbiota after chicken *E. tenella* infection. **(A)** Hierarchical clustering plots of eggNOG functional analysis. **(B)** Hierarchical clustering plots of KEGG functional analysis. Red and blue colors represent increased and decreased levels, respectively. JC, control group; JS, infected group.

To reveal the potential function of these differential microbiota, we performed functional pathway enrichment analysis. The eggNOG analysis results showed that the significantly enriched pathways mainly include signal transduction mechanisms and defense mechanisms, etc (**Figure 4A**). The KEGG enrichment analysis indicated that differential microbiota may be involved in pathways such as cell growth and death, immune system and immune diseases, and environmental adaptation (**Figure 4B**).

### 3.6 Transcriptome sequencing analysis of chicken cecum tissues after *E. tenella* infection

**Figure 5:**
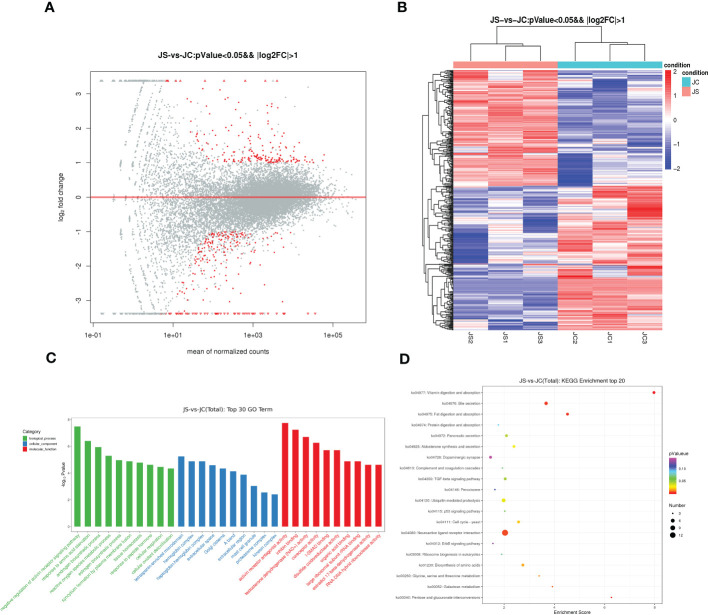
Transcriptome sequencing analysis of chicken cecal tissues after *E. tenella* infection. **(A)** The MA plot of differentially expressed mRNAs between JC and JS groups. Red dots represent differentially expressed genes, while gray dots represent genes with insignificant differences (196 up-regulated genes and 238 down-regulated genes). **(B)** Hierarchical clustering plots of differentially expressed mRNAs. Red and blue colors represent increased and decreased levels, respectively. **(C)** GO term analysis of differentially expressed mRNAs between JC and JS groups (Top 30). **(D)** KEGG pathway analysis of differentially expressed mRNAs between JC and JS groups (Top 20). The size of the circle represents the enriched number of differentially expressed genes and color represents significance. GO terms and KEGG pathways were considered to be significantly enriched based on a *P*-value <0.05. JC, control group; JS, infected group.

To investigate the effect of *E. tenella* infection on the host transcriptome, RNA-seq was carried out to detect differentially expressed mRNAs in chicken cecal tissues from the control and infection groups on day 4.5 pi, respectively. After removing the most discrete individuals from each group, the results showed that a total of 434 genes were differentially expressed in chicken cecal tissues after *E. tenella* infection, including 196 up-regulated genes and 238 down-regulated genes (**Figure 5A**). Hierarchical cluster analysis displayed that expression patterns of significantly differentially expressed mRNA between the control group and the treatment group were different (**Figure 5B**). The key differential expressed genes such as *GAMA*, *FABP1*, *F2RL1* and *RSAD2* may play an important role in host resistance to coccidiosis infection.

Moreover, to further reveal the function of differentially expressed genes, we performed both GO and KEGG pathway enrichment analysis. The top 30 significantly enriched GO terms were shown in **Figure 5C**. The results showed that the differentially expressed genes were mainly enriched in cellular oxidant detoxification, tissue homeostasis and negative regulation of activin receptor signaling pathway. The results of the significantly enriched top 20 KEGG pathways showed that the TGF-beta signaling pathway and ErbB signaling pathway were significantly enriched in JC and JS groups (**Figure 5D**).

### 3.7 Interaction analysis of gut microbiome and host transcriptome

**Figure 6:**
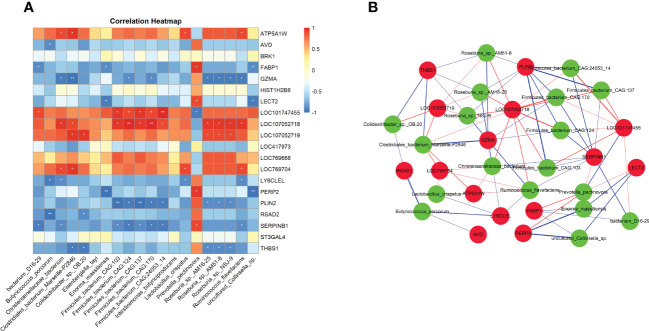
The interaction analysis of gut microbiome and host transcriptome. **(A)** Heatmap of Spearman’s correlation coefficients between differential microbiota and differential expressed mRNAs. Each row represents a differentially expressed mRNAs and each column represents a differentially microbiota. Red indicates positive correlations while blue indicates negative. The darker the color, the higher the correlation was. Spearman coefficient |r| > 0.8 and *P* < 0.05 were considered a significant difference. Correlation significance, *P < 0.05, **P < 0.01. **(B)** Association network of differential microbiota and differential expressed mRNAs. Red circles represent differentially expressed genes and green circles represent microbes. Red edges represent positive correlations and blue edges represent negative correlations. Edge thickness represents the strength of the correlation.

Through metagenomic sequencing analysis, we found that chicken *E. tenella* infection affect the changes in the abundance of cecal microbiota. In addition, we also identified a total of 434 differentially expressed genes in cecal tissues after *E. tenella* infection by using transcriptome sequencing. To explore the potential relationship between intestinal microbiota and host transcriptome after *E. tenella* infection, we use Spearman’s correlation coefficients to conduct the interaction analysis between differential microbiota and differentially expressed genes. As shown in the **Figures 6A, B**, *Prevotella pectinovora* has a significant positive correlation with *FABP1* (r=0.94, *P <*0.05), *LECT2* (r=0.88, *P <*0.05) and *SERPINB1* (r=0.88, *P <*0.05). *GZMA* has a significant negative correlation with *Roseburia* sp. *NSJ-9* (r=0.94, *P <*0.05) and *Roseburia* sp. *AM51-8* (r=0.95, *P <*0.05). *RSAD2* was significantly negatively correlated with *Butyricicoccus porcorum* (r=0.88, *P <*0.05) and *Colidextribacter* sp. *OB.20* (r=0.99, *P <*0.01). Notably, LOC107052718, an unknown gene, was significantly positively correlated with 11 cecal microbiota (*P <*0.05). SERPINB1 was only significantly positively correlated with *Prevotella pectinovora*, but had a significant negative correlation with the other 8 microbiota (*P <*0.05). These molecular markers may play an important role after *E. tenella* infection in chicken.

### 3.8 Correlation analysis of gut microbiome after *E. tenella* infection

**Figure 7:**
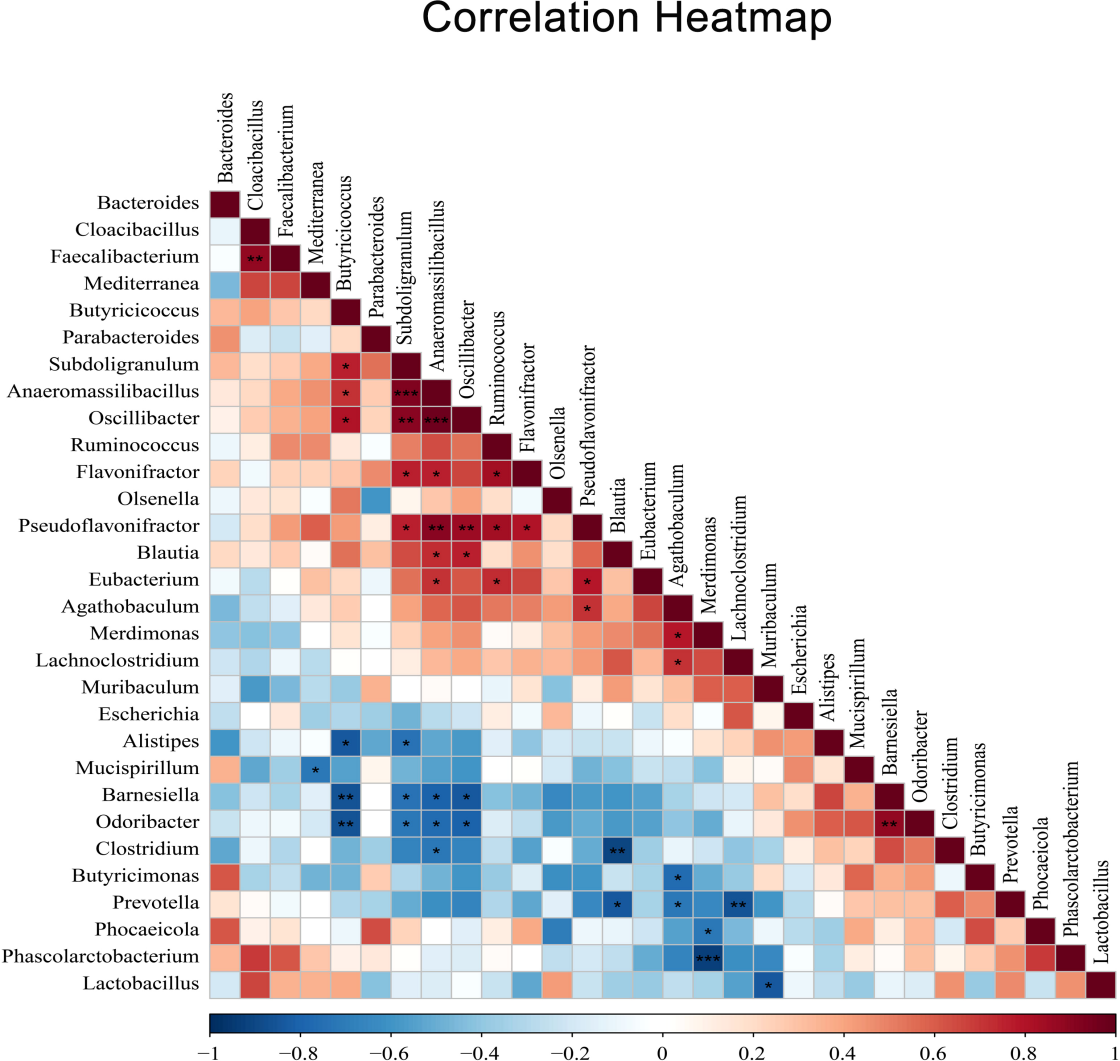
The heatmap of Spearman’s correlation coefficients between different dominant genus. Red indicates positive correlations while blue indicates negative. The darker the color, the higher the correlation was. Spearman’s correlations of different dominant genus were tested with a coefficient |r| > 0.8 and *P* < 0.05. Correlation significance, *P < 0.05, **P < 0.01, ***P < 0.001.

In addition, to reveal potential associations between cecal microbiota after coccidia infection, we selected the top 30 species in total abundance at the genus level and constructed the correlation analysis between dominant genus according to Spearman’s correlation coefficients (**Figure 7**). The results showed a significant negative correlation between *Alistipes* and *Butyricicoccus* (r=0.83, *P <*0.05) and a significant positive correlation between *Ruminococcus* and *Eubacterium* (r=0.73, *P <*0.05).

## 4 Discussion

The gastrointestinal microflora plays an important role in the nutrient absorption and immune protection process of poultry (Baldwin et al., 2018). Therefore, it is meaningful to study the effect of *E. tenella* infection on the cecal microbiota of chickens. In this study, metagenomic sequencing analysis of the cecal contents after *E. tenella* infection showed that *Bacteroidetes*, *Firmicutes*, *Proteobacteria*, and *Actinobactereia* were the most dominant phylum in the chicken cecal contents, which was consistent with previous studies consistent (Waite and Taylor, 2015; Zhou et al., 2020). Studies have shown that a gastrointestinal tract microbial of chickens with high diversity is expected to be more stable and healthier than a low diversity one (Clemente et al., 2012). In this study, compared with the control group, the proportion of *Firmicutes*, *Proteobacteria*, *Actinobactereia*, etc. at the phylum level in the infected group decreased, and the abundance of most genera such as *Faecalibacterium*, *Lactobacillus*, and *Mediterranea* also decreased. Therefore, *E. tenella* infection changed the diversity of cecal microbiota and affected the health of the host.

To screen out the important microbiota after *E. tenella* infection, we used Wilcoxon rank-sum test to analyze the significant difference of cecal microbiota between the JC and JS groups. The results showed that the abundance of *Lactobacillus* and *Roseburia* significantly decreased during *E. tenella* infection, while the abundance of *Prevotella pectinovora* significantly increased. In addition, we performed LEfSe analysis (an analysis method for biomarkers that find significant differences between different groups) on the cecal microbiota of the JC and JS groups. the results also showed that the abundance of *Lactobacillus* (includes *salivarius*, *aviarius*, *johnsonii*, and *crispatus*), *Roseburia sp* and *Faecalibacterium sp* decreased significantly, while the abundance of *Alistipes* increased after coccidia infection. *Lactobacillus* are an important component of the intestinal microbiota of mammals and birds, with functions such as resisting colonization by harmful bacteria, regulating inflammatory responses, and protecting the intestinal barrier (Zhao et al., 2021). Current evidence suggests that *Lactobacillus* play an important role in the anti-inflammatory immune response. Studies have shown that *Lactobacillus casei* and *Lactobacillus johnsonii* regulate the production of IL-12 in macrophages to adapt to infection by pathogenic bacteria (Teame et al., 2020). The peptidoglycan Ls33 purified from *Lactobacillus salivarius* also exerts anti-inflammatory effects by inducing the production of IL-10 (Fernandez et al., 2011). *Lactobacillus acidophilus* deficient in lipoteichoic acid downregulates the expression of IL-12 and TNF-a in dendritic cells and promotes IL-10 production to reduce intestinal inflammation (Mohamadzadeh et al., 2011). *Roseburia* is one of the commensal bacteria in the intestinal microbiota that produce short-chain fatty acids such as butyric acid, which maintain intestinal immunity and improve disease resistance (Tamanai-Shacoor et al., 2017). *Roseburia* has been shown to stimulate intestinal cells to secrete immune cytokines that promote the activation of regulatory T (Treg) cells (Nie et al., 2021). Butyrate produced by *Roseburia* promotes the production of IL-10, TGF-β, and IFN-r cytokines to influence the differentiation of Treg cytokines (Hegazy et al., 2017). Our previous study showed that Treg cells and related cytokines were up-regulated in cecum tissue after chicken coccidia infection, and are involved in the regulation of intestinal inflammation (Yu et al., 2021b). Therefore, coccidiosis infection causes structural destruction of intestinal tissues and changes in the intestinal microbiota, and butyrate produced by *Roseburia* may also be involved in the host’s inflammatory response by regulating the production of Treg cytokines. *Alistipes* are a relatively new microbiota isolated from the intestinal microbial community. Current research suggests that *Alistipes* reduce the inflammatory response in ulcerative colitis (UC) and inflammatory bowel disease (IBD) (Parker et al., 2020) and are also involved in cancer disease (Iebba et al., 2018). In poultry, *Alistipes* have been identified as producers of propionate in the chicken cecal microbiota (Polansky et al., 2015), and low levels of short-chain fatty acids such as butyric acid, propionic acid in the gastrointestinal system are associated with disease and inflammatory responses (Zaky et al., 2021). Our study showed the abundance of *Alistipes* in cecal microbiota increased significantly after *E. tenella* infection, which is consistent with the findings of Jebessa et al. (Jebessa et al., 2022). These evidence reveals that *Alistipes* may also be involved in the host immune response to chicken coccidia infection. Moreover, our study found that *Prevotella* are elevated after *E. tenella* infection in chickens. It was shown that elevated abundance of *Prevotella* enhances Th17-mediated mucosal inflammatory response and promotes Th17 cell immune factor production and neutrophil recruitment (Larsen, 2017). Th17 cell-mediated immune responses play an important role in the process of coccidia infection (Kim et al., 2012; Min et al., 2013), and thus elevated *Prevotella* may play an important role in immune response to chicken coccidia infection.

*Eimeria* infections have a serious impact on the physiological health of broiler chickens. The sporulated oocysts multiply in the cecum tissues and release a large number of sporozoites, which destroy intestinal epithelial cells and cause the blood vessels to rupture (Burrell et al., 2019; Madlala et al., 2021). Coccidia infection elicits a rapid response from the host immune system and some important genes play an important role in the inflammatory response to coccidial infection in chicken (Yu et al., 2021b). In this study, transcriptome sequencing of chicken cecal tissue after *E. tenella* infection was conducted to identify some important functional genes. A total of 434 genes were differentially expressed in cecal tissue, including immune-related genes such as *GAMA*, *FABP1*, *F2RL1* and *RSAD2*. GAMA interacts with the band 3 receptor to promote erythrocyte invasion by malaria parasites (Lu et al., 2022). A *Toxoplasma gondii* ortholog of plasmodium GAMA contributes to parasite attachment and cell invasion (Huynh and Carruthers, 2016). F2RL1 can regulate M1 macrophage polarization and inflammatory responses through the FOXO1 signaling pathway (Chen et al., 2019). Furthermore, high expression of F2RL1 in the intestine has been associated with intestinal inflammatory and oncogenic processes (Ke et al., 2020). Therefore, these genes related to immune may be involved in the host’s immune response against coccidioides. Functional enrichment analysis of these differentially expressed genes showed that the TGF-beta and ErbB signaling pathways were significantly enriched. TGF-beta signaling pathway was involved in host’s immune tolerance and liver fibrosis during *Echinococcus multilocularis* infection (Wang et al., 2013). Moreover, TGF-β1-induced Smad signaling pathway played a critical role in the activation of HSCs during liver fibrosis induced by *Clonorchis sinensis* infection (Yan et al., 2015). Studies have shown that *Cryptosporidium parvum* maintains intracellular survival by activating the host cellular EGFR (ErbB1)-PI3K/Akt signaling pathway (Yang et al., 2023). Therefore, these pathways may also play an important role in the process of coccidia infection.

To unravel the interactions between the gut microbiota and the host transcriptome after *E. tenella* infection in chicken. In this study, Spearman correlation coefficient was used to analyze the association of transcriptome and metagenomics. The results showed that *FABP1* was significantly associated with *Prevotella pectinovora*. FABP1 is a fatty acid binding gene that plays an important role in regulating intestinal fatty acid metabolic pathways. Studies have shown that FABP1 regulates key lipid-sensitive pathways in macrophages and thus participates in the intestinal inflammatory response (Levy et al., 2001). In addition, the expression of the *FABP1* gene is downregulated in poultry infected with *necrotizing enteriti*s (*NE*), which in turn reduces fatty acid utilization and intestinal immunity (Gharib-Naseri et al., 2021). Therefore, the results of our study suggest that *FABP1* and *Prevotella pectinovora* may be jointly involved in host immunity and metabolism-related processes following coccidial infection. In addition, *RSAD2* is significantly associated with *Butyricicoccus* and *Colidextribacter*. RSAD2 is necessary for dendritic cell maturation (Jang et al., 2018). Moreover, RSAD2 can regulate B cell activity through the NF-kB signaling pathway (Zhu et al., 2021). *Butyricicoccus* is also an important intestinal probiotic that has a positive effect in intestinal inflammatory diseases. Notably, SERPINB1 was only significantly positively correlated with *Prevotella pectinovora*, but had a significant negative correlation with the other 8 microbiota. SerpinB1, a protease inhibitor and neutrophil survival factor, is widely involved in the inflammatory response of diseases (Benarafa, 2011; Gong et al., 2011). Research by Zhao et al. (2014) shows that SerpinB1 regulates homeostatic expansion of IL-17+ γδ and CD4+ Th17 cells. The results of this study suggest that the expression of SerpinB1 may affect the changes of cecal microbiota during coccidia infection. LOC107052718, an unknown gene, was significantly positively correlated with 11 cecal microbiota. Although there is no report on the function of the LOC107052718 gene, our results reveal that LOC107052718 may play an important role in the infection of *E. tenella*, and the specific function and regulatory mechanism need further study.

In conclusion, the results of this study indicated that *E. tenella* infection significantly altered the abundance of chicken cecal microbiota and also affected the regulation of host genes. Moreover, we also revealed some potential relationships between differentially expressed genes and microbiota after coccidia infection. These results provide a reference for our understanding of the impact of coccidiosis on the host and the relationship between cecal microbiota and the host gene expression.

## Data availability statement

The datasets presented in this study can be found in online repositories. The names of the repository/repositories and accession number(s) can be found below: NCBI; PRJNA678759; PRJNA943112.

## Ethics statement

The animal study was reviewed and approved by the Animal Welfare Committee of Yangzhou University (permit number: SYXK (Su) IACUC 2012-0029).

## Author contributions

HY and GD designed the study and wrote the manuscript. HY, QW and JT performed the experiments, collected the data. HY and QW analyzed the transcriptome data and prepared the figures. LD provided technical support for the sequencing details. KX, TZ, GZ, HW and ZZ reviewed the manuscript. All authors contributed to the article and approved the submitted version.
