# Supplementary material for: Comprehensive analysis of gut microbiome and host transcriptome in chickens after Eimeria tenella infection
Source: Front Cell Infect Microbiol. 2023 Jun 6;13:1191939. doi: 10.3389/fcimb.2023.1191939 (PMC10279956; doi:10.3389/fcimb.2023.1191939)
Supplement: Supplementary file 1 [file DataSheet_1.docx]

Supplementary Material

Comprehensive Analysis of Gut Microbiome and Host Transcriptome in Chickens After *Eimeria tenella* Infection**Hailiang Yu^1^, Qi Wang^1^, Jianqiang Tang^1^, Liyue Dong^1^, Guojun Dai^1*^, Tao Zhang^1^, Genxi Zhang^1^, Kaizhou Xie^1^, Hongsheng Wang^2^, Zhenhua Zhao^3^**

*** Correspondence:** Guojun Dai. daigj@yzu.edu.cn

# Supplementary Data


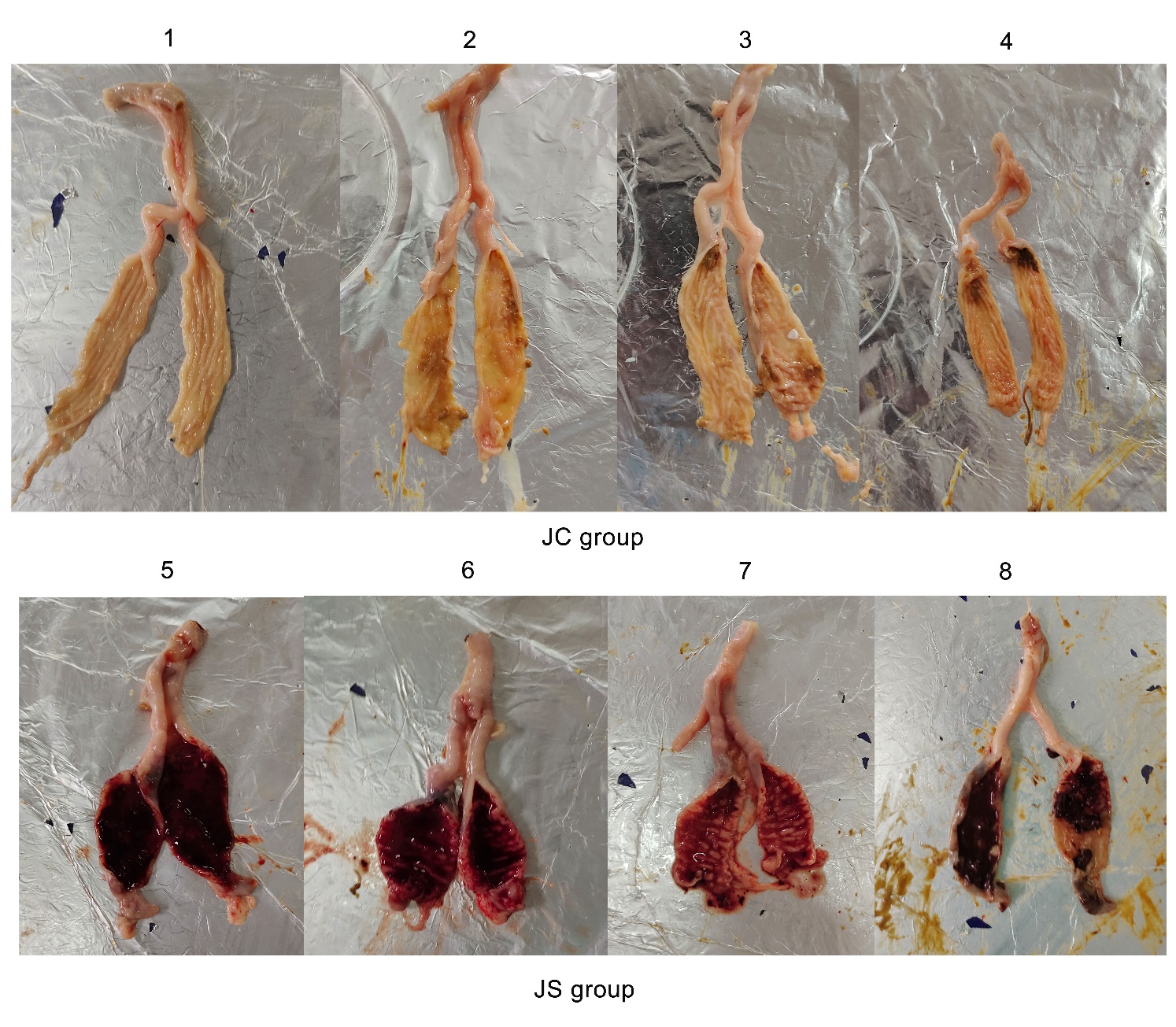


**Supplementary Figure 1: Images of cecal tissues of chickens at 4.5 days after *E. tenella* infection*.*** (1-4), the cecal tissues of chickens in JC group. (5-8), the cecal tissues of chickens in JS group.


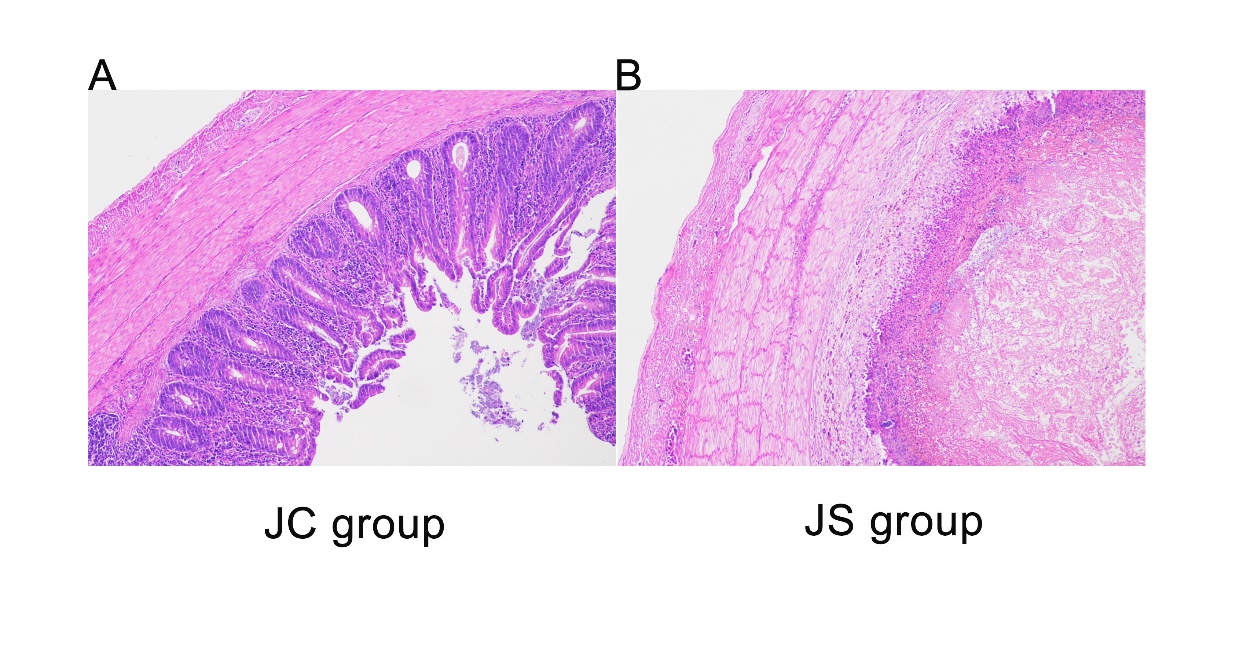


**Supplementary Figure 2: Histological and pathological images of cecal tissues of chickens at 4.5 days after *E. tenella* infection.** (A), the cecal tissues of chickens in JC group. (B), the cecal tissues of chickens in JS group.


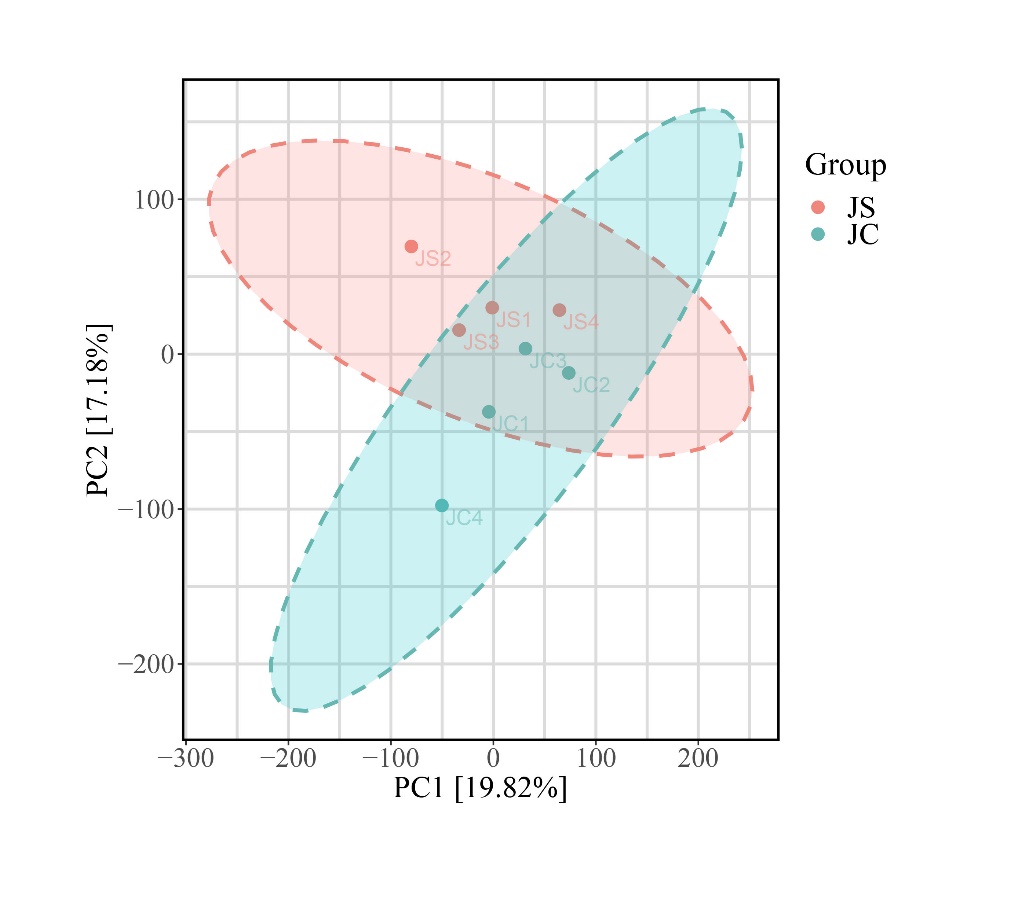


**Supplementary Figure 3: Principal component analysis (PCA) of cecal microbiota composition between JC (blue circle) and JS groups (red circle).** JC, control group; JS, infected group.


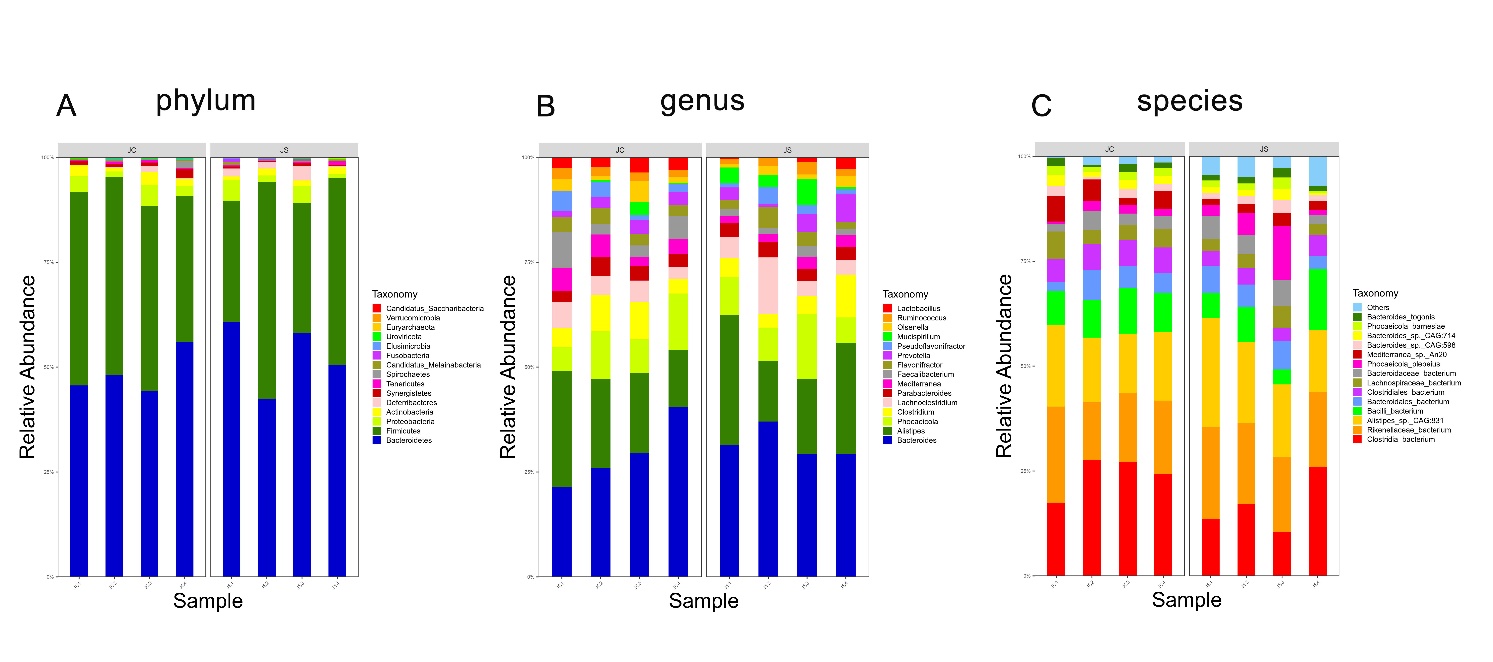


**Supplementary Figure 4: Comparison of microbial community composition between JC and JS groups.** (A), phylum. (B), genus. (C), species. JC, control group; JS, infected group. Each column represents a sample.
